# Capsular Switching and ICE Transformation Occurred in Human *Streptococcus agalactiae* ST19 With High Pathogenicity to Fish

**DOI:** 10.3389/fvets.2018.00281

**Published:** 2018-11-13

**Authors:** Rui Wang, Liping Li, Ting Huang, Weiyi Huang, Aiying Lei, Ming Chen

**Affiliations:** ^1^Guangxi Key Laboratory for Aquatic Genetic Breeding and Healthy Aquaculture, Guangxi Academy of Fishery Sciences, Nanning, China; ^2^Institute of Animal Science and Technology, Guangxi University, Nanning, China

**Keywords:** GBS, ST19, capsular switching, ICE, cross-species infection

## Abstract

Although *Streptococcus agalactiae* (GBS) cross-infection between human and fish has been confirmed in experimental and clinical studies, the mechanisms underlying GBS cross-species infection remain largely unclear. We have found different human GBS ST19 strains exhibiting strong or weak pathogenic to fish (sGBS and wGBS). In this study, our objective was to identify the genetic elements responsible for GBS cross species infection based on genome sequence data and comparative genomics. The genomes of 11 sGBS strains and 11 wGBS strains were sequenced, and the genomic analysis was performed base on pan-genome, CRISPRs, phylogenetic reconstruction and genome comparison. The results from the pan-genome, CRISPRs analysis and phylogenetic reconstruction indicated that genomes between sGBS were more conservative than that of wGBS. The genomic differences between sGBS and wGBS were primarily in the Cps region (about 111 kb) and its adjacent ICE region (about 106 kb). The Cps region included the entire *cps* operon, and all sGBS were capsular polysaccharide (CPS) type V, while all wGBS were CPS type III. The ICE region of sGBS contained integrative and conjugative elements (ICE) with IQ element and erm(TR), and was very conserved, whereas the ICE region of wGBS contained ICE with mega elements and the variation was large. The capsular switching (III–V) and transformation of ICE adjacent to the Cps region occurred in human GBS ST19 with different pathogenicity to fish, which may be related to the capability of GBS cross-infection.

## Introduction

*Streptococcus agalactiae*, also known as Group B *Streptococcus* (GBS), is considered as a colonizing agent of humans and the major cause of neonatal pneumonia, meningitis and sepsis, and the frequency of invasive infection in adults has also increased within the past two decades (1). Animal infection studies have demonstrated that human GBS can infect fish (2, 3), while the clonal complex (CC) 23 clinical isolates, which belong to primarily human isolates cluster, have been isolated from aquatic animals (4). In recent years, GBS has become an emerging pathogen in aquatic environments and infects many species of culture fish, causing septicemia and meningoencephalitis, especially on tilapia farms (5). Studies show that consumption of fish has been associated with an increased risk of GBS colonization in people, as well as the spread of fish GBS in humans (6, 7). In 2015, an outbreak of infection with GBS associated with raw freshwater fish consumption was reported, which affected more than 200 patients in Singapore (8). GBS spread quickly through the fish and water, which can cause a large area of Streptococcal outbreak in aquaculture in a short time (9). Therefore, the cross-species infection of GBS between human and fish has become a serious threat to human health, but the mechanism of GBS cross-species infection remains largely unknown.

CC19, including its namesake sequence type (ST) 19, is at the center of the GBS population according to the extent of highly conserved regions between the different CCs (10), which have been linked to carriage or disease in people and cattle (11, 12). Nevertheless, none isolate of CC19 has been reported in fish. We used invasive human GBS ST19 (28 strains) to infect tilapia, and found that 17 strains were strong pathogenic to tilapia (sGBS, the mortality rate: 70–100%), while 11 strains showed weak pathogenicity to tilapia (wGBS, the mortality rate: 0–30%) (13). In the present study, the genomic difference analysis was performed between sGBS and wGBS to explore the mechanisms related to the capability of GBS cross-species infection.

## Materials and methods

### Genome sequencing, assembly, and annotation

Twenty-nine invasive GBS ST19 strains were obtained from 29 patients in five hospitals of three cities in Guangxi Province, and the details of the strains information and the isolation procedure were reported in our previous article (13). The genomes of 22 strains including sGBS (11 strains) and wGBS (11 strains) were sequenced using the Illumina HiSeq2000 sequencing platform and the results were assembled using the ABySS program 1.9.0 (14). The assembly coverage was not <250×, the number of contigs was 44–118, average N50 value was 94,316, and average number of coding cequences was 2,233. The whole genome shotgun (WGS) sequences of 22 ST19 GBS strains have been deposited in GenBank. The genome contigs of sGBS strain LZF001 and wGBS strain LZF006 were rearranged using the genome of GBS 2603V/R strain as the reference genome by Mauve (15). The CPS type, ST, CC, host and GenBank accession number of the sequenced strains and 2603V/R were listed in Additional file 1. The assembled genome was uploaded to the RAST website and was annotated by using the RASTtk program on line (16).

### Pan-genome analysis of ST19 GBS

The pan genome analysis was performed for 22 ST19 GBS strains using Roary with a blast identity cutoff of 97% (17). The visualization of the pan-genome was performed by Anvi'o 2.4.0 (18).

### CRISPRs analysis, and phylogenetic reconstruction

The clustered regularly interspaced short palindromic repeats (CRISPRs) of the strains were identified by CRISPRs finder and CRISPR recognition tool (CRT) (19, 20), each unique spacer of the CRISPRs was numbered manually, and further optimized based on the report from Lier et al. (21). The orthologous protein searches were performed using OrthoMCL 2.0.9 (22). Multiple sequence alignment of single copy homologous protein sequences was performed by MAFFT and the poorly aligned positions and divergent regions of the alignment were removed. The maximum likelihood (ML) estimation of phylogenetic trees and model parameters were performed by PhyML program, and the optimal amino acid substitution model was obtained by comparing AIC and BIC scores (23). RaxML software was used to construct the ML based phylogenetic tree with 1,000 bootstrap replications (24). The visualization of the phylogenetic tree was performed by FigTree 1.4.3 (http://tree.bio.ed.ac.uk/software/figtree/).

### Genomic comparison

The genome comparative analysis was performed for 12 ST19 GBS representative strains, including 6 sGBS strains and 6 wGBS strains. The common and unique genes from annotated genomes of strains were search and compared by using the sequence based genomic comparison tool provided by SEED viewer (25). Briefly, wGBS LZF006 and sGBS LZF001 were used as the reference genome, respectively, and the genomes of other 10 strains were aligned to the reference genome. The result listed the genes of the reference organism in chromosomal order and displayed hits on the comparison organisms accordingly. The evolutionary analysis for homologous proteins from GBS *cps* operon was performed by using the above-mentioned method. The CPS type, ST, CC, host and GenBank accession number of strains involved in evolutionary analysis were listed in Supplementary Table 1.

## Results

### Analysis of the pan-genome

**Figure 1:**
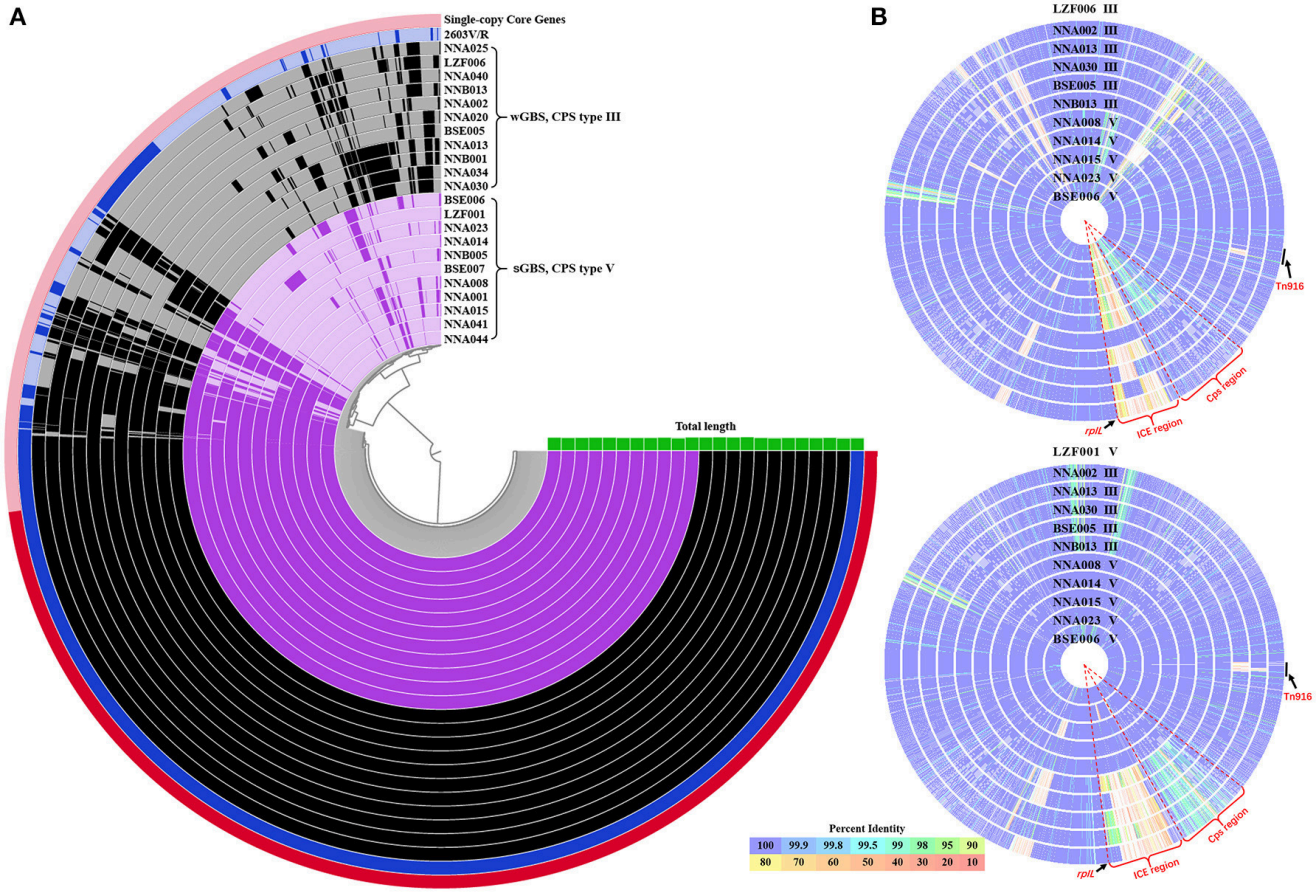
The pan-genome and comparison of sGBS and wGBS genomes. **(A)** Anvi'o pangenome visualization of sGBS and wGBS genomes. The red part in outer ring showed single-copy core genes. The genomes of sGBS and wGBS were shown in purple and black, respectively, and the 2603V/R genome was shown in blue, the green histogram meaned genome length. **(B)** Comparison of genomes of sGBS and wGBS. The LZF006 (wGBS) genome and the LZF001 (sGBS) genome were served as the reference, respectively, and other genomes were aligned to the reference genome; each circle represented a different genome, the strain name and CPS type were labeled on each circle. The circle of reference genome was not shown; the color bar on the bottom indicated the percentage of protein sequence identity against the reference genome. The relevant areas and locus were indicated by black arrows in the figure.

The average genome size of 22 GBS ST19 strains was 2.24 Mb, with an average gene density of 1 gene per kb. The GC content of genome from 11 wGBS strains was 35.70%, and the average number of genes was 2,247 with an average length of 884 bp for each gene. The GC content of genome from 11 sGBS strains was 35.66%, and the average number of genes was 2,208 with an average length of 902 bp for each gene. The total number of genes from 11 wGBS strains was 2,753, and the proportion of core genes and accessory genes was 67.09% (1,847/2,753) and 32.91% (906/2,753), respectively. The total number of genes from 11 sGBS strains was 2,713, and the proportion of core genes and accessory genes was 70.59% (1,915/2,713) and 29.41% (798/2,713), respectively. The total number of genes from 22 GBS strains was 3,223, among which 1,758 were core genes (54.55%) and 1,465 were accessory genes (45.45%). Visualizations of the results was shown in Figure 1A.

### CRISPRs analysis and phylogenetic reconstruction

**Figure 2:**
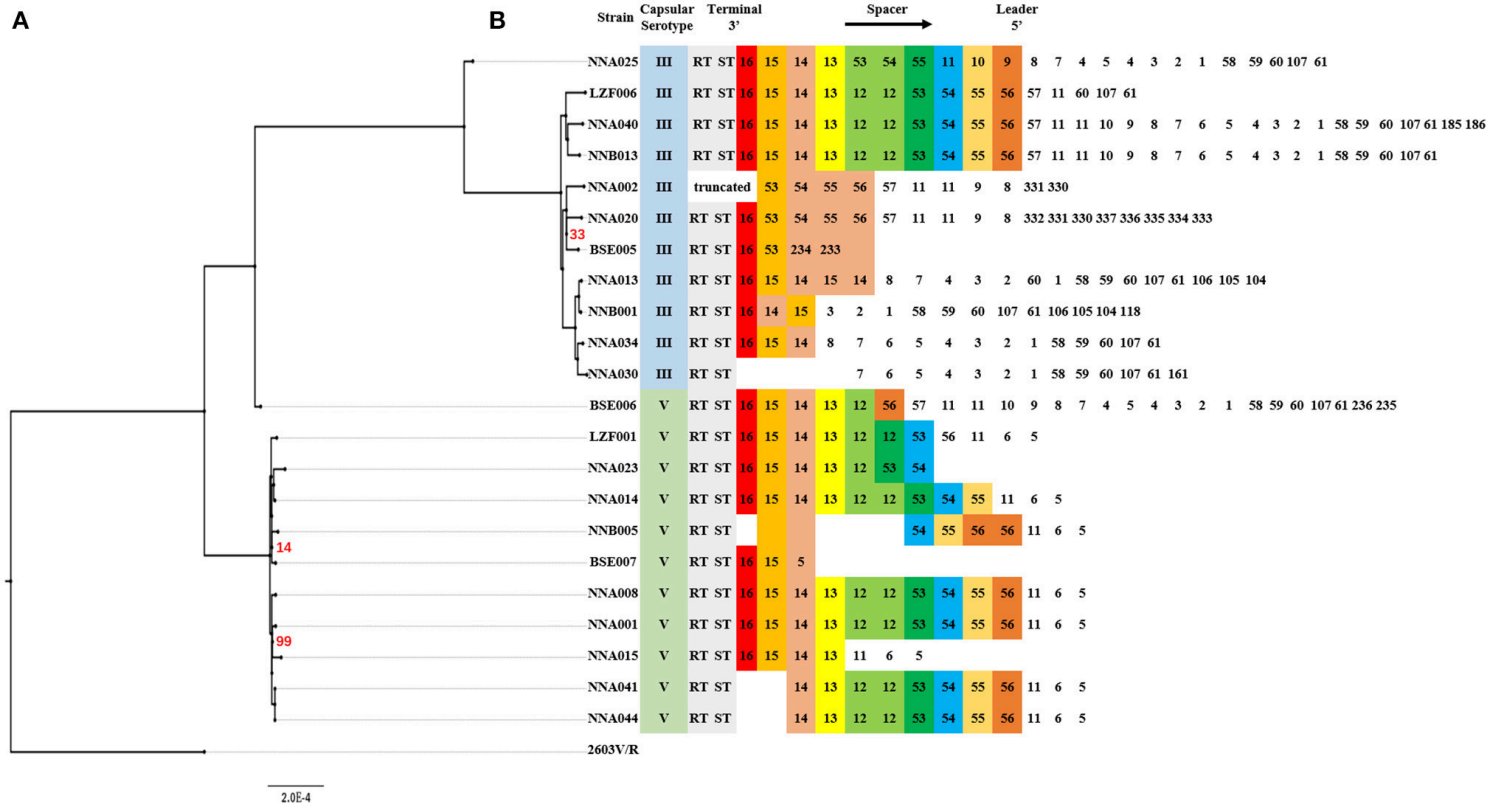
Comparative analysis of systematic evolution and CRISPRs structure of ST19 GBS strains. **(A)** A Maximum Likelihood phylogenetic tree based on 1,707 single copy orthology clusters of the 23 strains, and 2603V/R strain was used as outgroup strain. The bootstrap confidence value which was 100 was not shown in the figure. **(B)** CRISPRs structure comparison. Direct repeat sequence was not included; only RT, ST, and spacers were represented. The spacers were numbered, and the same number highlighted with same color indicated that the spacer sequence was the same.

CRISPRs analysis and phylogenetic reconstruction of ST19 GBS were shown in Figure 2. The spacers numbered 11–16 and 53–56 were more conserved spacers in wGBS strains. Generally, the number and type of spacers in wGBS strains were more than that in sGBS strains. The phylogenetic analysis showed that the evolution distance between wGBS strains was farther than that between sGBS strains. The sGBS strain BSE006 was clustered with the wGBS strains, and the CRISPRs structure of BSE006 was also similar to the wGBS strains.

### Genome comparison

The genome comparison between the wGBS strains and the sGBS strains was shown in Figure 1B. The regions with common variation in genome were mainly located at the Cps region containing complete *cps* operon and ICE region.

### CPs region

**Figure 3:**
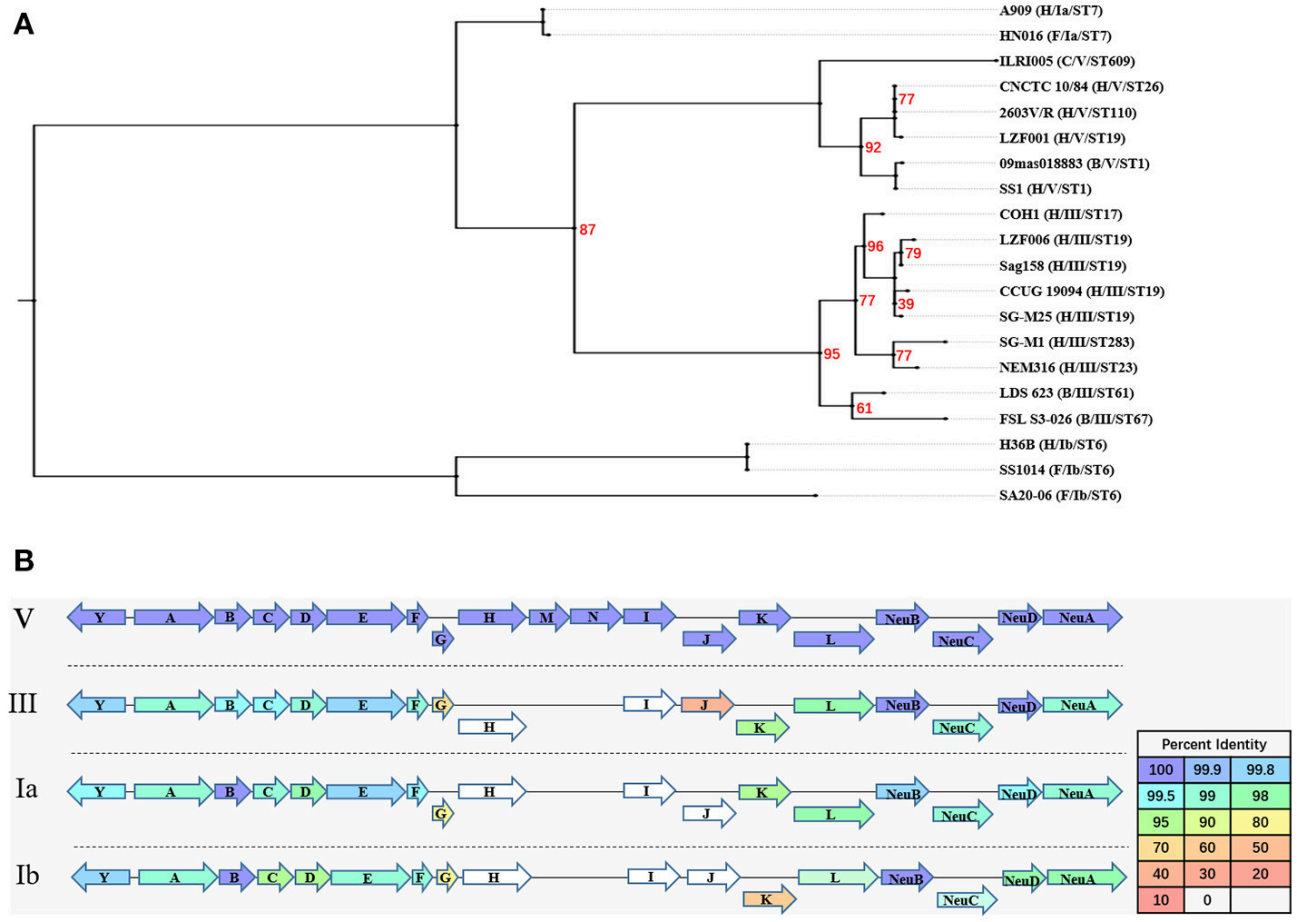
The evolutionary analysis of homologous proteins in *cps* operon and their structure. **(A)** A Maximum Likelihood phylogenetic tree based on 12 single copy orthology clusters of *cps* operon from 20 strains. The bootstrap confidence value which was 100 was not shown in the figure. **(B)** The *cps* operon structure of GBS serotypes Ia, Ib, III, and V. The *cpsY, cpsA-cpsL, NeuA-NeuD* genes were labeled with arrows, and the arrow showed the direction of transcription. The different color of arrow indicated the percent identity of the encoded protein.

The results of the genome comparison showed that a large fragment of about 111 kb was involved in the variation of capsule locus region (Figure 1B). This large fragment was flanked by two transposons, involving 109 genes including 19 genes on *cps* operon. Except the deletion mutation in the *cps* operon, the percentage of protein sequence identity of the other genes was 97.04–100 against the reference genome. The analysis of *cps* operon showed that all sGBS were CPS type V, while all wGBS were CPS type III. The *cps* operon structures of CPS types Ia, Ib, III, and V were shown in Figure 3. All *cps* operons from Ia, Ib, and III did not contain *cpsM* and *cpsN* genes. There were different degree of variation in the *cps* operons in Ia, Ib, III, and V, while the *cpsB* gene sequence of CPS type V was exactly same as Ia and Ib. The phylogenetic trees for the homologous proteins of *cps* operon from different CPS types were further constructed and it was shown that the different ST strains from the same CPS type were clustered into one branch. The human Ia strain and the fish Ia strain, as well as the human Ib strain and the fish Ib strain were clustered into one branch.

### ICE region

A total of 127 genes were involved in the ICE region of the sGBS strains and were identified with characteristic of two putative ICE, including ICE*Sag*LZF001IQ with IQ element and ICE*Sag*LZF001erm with *erm* fragment. IQ element carried two associated genes *mef* (I) and *catQ*, which were macrolides resistant gene, and chloramphenicol resistance gene of *Streptococcus*, respectively (26–28). *erm* fragment carried *Erm*(TR), which was an *erm*(A) subclass gene discovered by Seppala et al. (29) and has become an increasingly popular macrolides resistance gene of *Streptococcus* (30).

**Figure 4:**
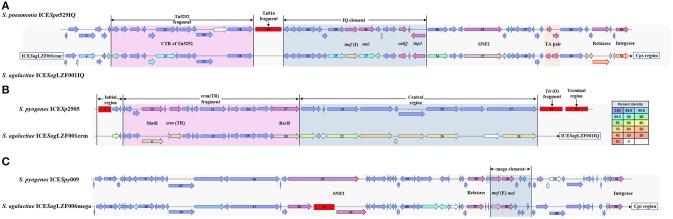
Gene organization of putative integrative conjugative element ICE*Sag*LZF001IQ, ICE*Sag*LZF001erm, and ICE*Sag*LZF006mega. **(A)** Schematic representation of ICE*Sag*LZF001IQ with ICE*Spn*529IQ as the genetic reference. **(B)** Schematic representation of ICE*Sag*LZF001erm with ICE*Sp*2905 as the genetic reference. **(C)** Schematic representation of ICE*Sag*LZF006mega with ICE*Spy*009 as the genetic reference. The arrows indicated the direction of the transcription. The color of the arrow was correlated with the percent identity. The arrows with red border were annotated the gene information. The deletion regions were shown with red rectangle. The number in the arrow indicated the serial number of ORFs, and the number in the red square indicated the number of ORFs within the fragment.

ICE*Sag*LZF001IQ was 28,763 bp in size, had a G + C content of 36.12% and consisted of 54 putative open reading frames (ORFs). Its genetic organization was characterized using ICE*Spn*529IQ (GenBank accession: HG965092) (31) as the genetic reference (Figure 4A). As in ICE*Spn*529IQ, the recombination related proteins, such as integrase, relaxase, and recombinase were encoded, an IQ elements and a TA pair were contained in ICE*Spn*529IQ. The products of *mef* (I) and *catQ* displayed 99 and 100% protein identity to the corresponding amino acid sequences of ICESpn529IQ, respectively. The TA pair coding for a toxin-antitoxin (TA) system includes the toxin gene *pezT* encoding a protein that is homologous to the zeta toxin, and the antitoxin gene *PezA*. The TA pair of ICE*Sag*LZF001IQ showed 100% protein identity to the corresponding TA pair of ICESpn529IQ. The details of the ICE region comparison between ICE*Sag*5H001IQ and ICE*Spn*529IQ were shown in Supplementary Table 2.

ICE*Sag*LZF001erm was 36,531 bp in size, had a G + C content of 32.20% and consisted of 28 putative ORFs. Its genetic organization was characterized using ICE*Sp*2905 (GenBank accession: FR691055) (30) as the genetic reference (Figure 4B). As in ICE*Sp*2905, an initial region, entire erm(TR) fragment, and central region were contained in ICE*Sag*LZF001erm. The erm(TR) fragment contained *emr* (TR) encoding erythromycin resistance methylase and *MatE* gene encoding multi antimicrobial extrusion protein. Except *MatE, erm*(TR), and recombinase (*RecB*), the products of the other 12 genes in erm(TR) fragment showed 100% protein identity to the corresponding amino acid sequences of ICE*Sp*2905. The details of the ICE region comparison between ICE*Sag*LZF001erm and ICE*Sp*2905 were shown in Supplementary Table 3.

The ICE region of the wGBS strains included 102 genes and was identified with the characteristic of one putative ICE, ICE*Sag*LZF006mega, with mega element. The mega element carried macrolides resistant gene *mef* (E) (26, 27). ICE*Sag*LZF006mega was 106,400 bp in size, had a G + C content of 36.09% and consisted of 99 putative ORFs. Its genetic organization was characterized using ICE*Spy*009 (GenBank accession: KU056701) (26) as the genetic reference (Figure 4C). As in ICE*Spy*009, intergrase, relaxase, SNF2, and other ICE recombination related proteins were encoded, and a mega element containing *mef* (I) and *mel* was carried in ICE*Sag*LZF006mega. In addition, the SNF2 gene of ICE*Sag*LZF006mega was truncated and a fragment containing 38 ORFs was inserted. The details of the ICE region comparison between ICE*Sag*LZF006mega and ICE*Spy*009 were shown in Supplementary Table 4.

## Discussion

The serotype of the strains was confirmed by molecular serotyping in our other study (13). In this study, we have found that the capsular switching (III–V) and transformation of ICE adjacent to the Cps region occurred in human GBS ST19 with different pathogenicity to fish, which may be related to the capability of GBS cross-species infection.

Although a variety of DNA-based GBS population diversity analysis have clearly indicated the existence of GBS capsular switching (32–35), there was no report of capsular switching analysis based on comparative genomics and the relationship with cross infection. In the present study, the results of the pan-genome, CRISPRs analysis and phylogenetic reconstruction of ST19 GBS all suggested that CPS type V sGBS emerged through capsular switching from CPS type III and BSE006 seemed be an intermediate strain in the switching. The whole genome comparative analysis showed that the high variations were observed not only in cps operon between CPS type V and III strains, but also in the flanking regions of cps operon, suggesting that this region likely underwent large fragment recombination. In addition, a capsular switching from CPS type III–IV within CC17 GBS also occurred due to the exchange of a 35.5 Kb DNA fragment containing the entire *cps* operon (32), indicating that the recombination of large DNA fragments including *cps* operon is an important way of GBS capsular switching. The major CPS types of GBS that currently infect fish are CPS types Ia and Ib (36). In this study, the results showed the *cpsB* gene in the *cps* operon of the ST19 CPS type V was identical to that of the Ia and Ib. The capsules can affect the adherence of GBS to fish intestinal epithelial cells (37), and CpsB participates in determining the length and adhesion of GBS capsules (38). In summary, the capsules, especially CpsB, may play an important role in GBS virulence and cross-species infection.

Clustered regularly interspaced short palindromic repeats (CRISPRs) are a bacterial adaptive immune defense mechanism for defending against foreign nucleic acids, such as ICE, plasmids, and phages (39). CRISPR arrays are composed of a peculiar family of DNA repeats which are usually multiple, non-contiguous, direct DNA repeats interspaced by non-repetitive nucleotides (spacer) (40). When foreign nucleic acids invades bacteria, the CRISPRs integrate and save the intruding gene fragment (spacer). Under the re-invasion of the same genes, mediated by specific RNA, CRISPRs, and CRISPR-associated proteins (Cas proteins) will cut and destroy the invading foreign nucleic acids (41). The repeats are highly conserved within a given CRISPR array, and the spacers are correspond to segments of captured viral and plasmid sequences in most cases (42). The differences of the CRISPRs structure implied different capabilities between wGBS and sGBS to defend against foreign nucleic acids, which may affect their adaptive capacity in different host. The strains of sGBS derived from a recent common ancestor as the CRISPRs structure of sGBS was more consistent than that of wGBS. The mechanism of the recombination of large DNA fragments, capsular switching and differences of CRISPRs structure are not completely understood. Capsular switching and different CRISPRs structure are probably driven by the host immune response and the living conditions of the bacteria. Therefore, study of characteristics of immunity and *in vivo* environment of the hosts, which sGBS and wGBS were isolated from, would help to clarify the relevant mechanism.

ICE are widely distributed mobile DNA elements that can be vertically propagated by integrating into the host genome or can be transferred horizontally by excision and transfer of elements to new hosts. We identified an ICE variant region associated with GBS ST19 capsular switching through the whole genome comparison. With the transformation of ST19 GBS CPS type from III to V, the ICE in this region was also transformed from the original ICE to the new one. Similarly to the capsular switching, it is possible that the ICE transformation is a result from the adaption of bacteria to the host immunity and environment. In CPS type III strains, the region is located at the 3′ terminus of ST19 GBS Cps region, and contains an ICE carrying mega element. In contrast, this region in CPS type V contains two ICEs, carrying IQ element and the erm (TR) fragment, respectively. The resistant genes in mega element, IQ element, and erm (TR) fragment are all macrolides resistance genes (26, 27, 29, 30). The effect of different macrolides resistance genes on GBS adaptability remains to be further studied.

The large-scale aquiculture of fish and the high carrying rate of GBS in human result in an increased frequency of genome exchange intra- and inter-species with different hosts, which increases the risk of producing cross-species infection strains or highly adaptable strains. Our data indicated that capsular switching and ICE transformation might improve the ability of GBS cross-species infection, which resulted in an increased difficulty for preventing GBS diseases. It should be emphasized that great attention should be paid to monitor the colonization and disease of GBS in practitioners from fish farms, and study the associations among GBS strains with different hosts, which will be important for the prevention and control of GBS disease.

## Author contributions

RW and LL analyzed data and wrote the manuscript; TH, WH, and AL performed experiments and analyzed data; MC conceived and designed the study. All authors reviewed and approved the manuscript.

### Conflict of interest statement

The authors declare that the research was conducted in the absence of any commercial or financial relationships that could be construed as a potential conflict of interest.
