# Supplementary material for: Capsular Switching and ICE Transformation Occurred in Human Streptococcus agalactiae ST19 With High Pathogenicity to Fish
Source: Front Vet Sci. 2018 Nov 13;5:281. doi: 10.3389/fvets.2018.00281 (PMC6242859; doi:10.3389/fvets.2018.00281)
Supplement: Supplementary file 1 [file Table_1.DOC]

**Supplementary Table 1 GBS strains included in this study.**

| **Strain** | **CPS type** | **Sequence type (ST)** | **clonal complex (CC)** | **Host** | **GenBank accession number** |
| --- | --- | --- | --- | --- | --- |
|
| BSE005(wGBS) | Ⅲ | 19 | 19 | Human | NAWG00000000 |
| LZF006(wGBS) | Ⅲ | 19 | 19 | Human | NAYV00000000 |
| NNA002(wGBS) | Ⅲ | 19 | 19 | Human | NAYQ00000000 |
| NNA013(wGBS) | Ⅲ | 19 | 19 | Human | NAYF00000000 |
| NNA020(wGBS) | Ⅲ | 19 | 19 | Human | NAXS00000000 |
| NNA025(wGBS) | Ⅲ | 19 | 19 | Human | NAXN00000000 |
| NNA030(wGBS) | Ⅲ | 19 | 19 | Human | NAXI00000000 |
| NNA034(wGBS) | Ⅲ | 19 | 19 | Human | NAXD00000000 |
| NNA040(wGBS) | Ⅲ | 19 | 19 | Human | NAWX00000000 |
| NNB001(wGBS) | Ⅲ | 19 | 19 | Human | NAXY00000000 |
| NNB013(wGBS) | Ⅲ | 19 | 19 | Human | NAWA00000000 |
| BSE006(sGBS) | Ⅴ | 19 | 19 | Human | NAWF00000000 |
| BSE007(sGBS) | Ⅴ | 19 | 19 | Human | NAWE00000000 |
| LZF001(sGBS) | Ⅴ | 19 | 19 | Human | NAYZ00000000 |
| NNA001(sGBS) | Ⅴ | 19 | 19 | Human | NAYR00000000 |
| NNA008(sGBS) | Ⅴ | 19 | 19 | Human | NAYK00000000 |
| NNA014(sGBS) | Ⅴ | 19 | 19 | Human | NAYE00000000 |
| NNA015(sGBS) | Ⅴ | 19 | 19 | Human | NAYD00000000 |
| NNA023(sGBS) | Ⅴ | 19 | 19 | Human | NAXP00000000 |
| NNA041(sGBS) | Ⅴ | 19 | 19 | Human | NAWO00000000 |
| NNA044(sGBS) | Ⅴ | 19 | 19 | Human | NAVZ00000000 |
| NNB005(sGBS) | Ⅴ | 19 | 19 | Human | NAWU00000000 |
| 2603V/R | V | 110 | 19 | Human | NC_004116.1 |
| A909 | Ia | 7 | 7 | Human | CP000114.1 |
| HN016 | Ia | 7 | 7 | Fish | CP011325.1 |
| ILRI005 | IV | 609 | Unknown | Camel | NC_021486.1 |
| CNCTC 10/84 | V | 26 | 26 | Human | CP006910.1 |
| 09mas018883 | V | 1 | 1 | Cattle | HF952104.1 |
| SS1 | V | 1 | 1 | Human | CP010867.1 |
| COH1 | III | 17 | 17 | Human | HG939456.1 |
| Sag158 | III | 19 | 19 | Human | CP019979.1 |
| CCUG 19094 | III | 19 | 19 | Human | ALQK01000030 |
| SG-M25 | III | 19 | 19 | Human | CP021867.1 |
| SG-M1 | III | 283 | 10 | Human | CP012419.2 |
| NEM316 | III | 23 | 23 | Human | AL732656.1 |
| LDS 623 | III | 61 | 61 | Cattle | ANEX01000127 |
| FSL S3-026 | III | 67 | 67 | Cattle | AEXT01000007 |
| H36B | Ib | 6 | 7 | Human | AAJS01000001 |
| SS1014 | Ib | 6 | 7 | Fish | CAQC01000077 |
| SA20-06 | Ib | 553 | 552 | Fish | CP003919.2 |
